# Transcendent and Transcendental Time Perspective Inventory

**DOI:** 10.3389/fpsyg.2018.02677

**Published:** 2019-01-10

**Authors:** Celina Timoszyk-Tomczak, Beata Bugajska

**Affiliations:** Faculty of Humanities, Institute of Psychology, University of Szczecin, Szczecin, Poland

**Keywords:** transcendental time perspective, transcendent time perspective, gerotranscendence, future, time perspective

## Abstract

The purpose of this paper is to propose a tool to examine the transcendent and transcendental time perspective (TTTP). The inspiration to develop the scale were Philip G. Zimbardo and John N. Boyd studies, as well as by Lars Tornstam’s gerotranscendence theory and own research. The analysis of life from death to eternity is an interesting, heterogeneous and difficult subject of study. The proposed TTTP inventory can be utilized to investigate the future that extends beyond the frames of a personal time perspective, beyond the individual’s death as well as beyond the recognized, standard ways of understanding oneself, other people and the world. The inventory refers to changes of quantitative and qualitative nature relating to what is going to happen. It is composed of two sub-scales: the transcendental future and the transcendent future. The paper outlines the psychometric values of the qualities of the inventory, its validity and accuracy based on such indicators as the discriminative of items, the Cronbach alpha index for each of the sub-inventories and the exploratory factor analysis. The study findings come from analyses conducted on a group of 211 elderly subjects (the average age of 65; 70% women, 30% men). A confirmatory factor analysis was also conducted on a group of 238 elderly subjects (the average age of 66; 69% women, 28% men, 3% no gender data available). Additionally, the paper presents data on the accuracy of the external scale. The data are interpreted in the light of the time perspective theory as well as the existing studies.

## Introduction

The questions of time and timing are a subject of interest in many fields of science. Time is one of the most fundamental and universal categories by means of which people can grasp the reality and which make it comprehensible. Time is an indispensable element of individual and social experience (Bokszański et al., 1998). The studies on the psychology of time date back to the beginning of the 20th century. The dynamics of these studies varied over years from initial fascination to the loss of interest to revival (Hancock and Block, 2012). The psychologists usually analyze psychological time, which people sometimes perceive subjectively. This means that psychological time is personal, biased and conditioned by one’s experience. The non-objectivity of time is a function of human activity and of related experience (Bakiera and Stelter, 2011). The analyses of psychological time encompass a wide range of subjects from biological rhythms to the perception of diverse aspects of time to culture change and its rate. As a result of this diversity of subjects many models have been developed of psychological time seen from different angles: bio-psychological, behavioral, cognitive, developmental, psychoanalytical or socio-psychological (Block and Grondin, 2014). Within the framework of the studied into psychological time the researchers examine such constructs as time perspective, temporal orientation, attitude toward time, time competences, time pressure, time structuring and planning, time awareness, the telic nature of time and time management, the passage of time or time in interpersonal relations (Nuttin, 1985; Zaleski, 1994; Uchnast, 2006; Zimbardo and Boyd, 2008).

Another term associated with psychological time is mental travel hypothesized by Tulving (1972, 1985, 2002). He considered episodic memory to be a basis for re-living the past and pre-living the future. Mental travel may envelop the spans of time beyond individual existence. An individual can travel in time to moments prior to their birth or after their death (Tulving and Kim, 2007). Traveling to these time moments comprises the personal, generational and metaphysical contexts and is connected with the stress caused by such ultimate experience as one’s own death (Suddendorf and Corballis, 2007). Time traveling which transcends the time limits of one’s own life cannot be compared to traveling in time within the limits of existence. It is based on the belief in the existence of something beyond death and related to confronting oneself with the end of one’s own existence.

In reference both to psychological time and to the ability to travel mentally in time, the future becomes particularly important. The future is a domain where the individual is motivated and which influences their behavior and activity (Nuttin, 1985). It encompasses what is unexpected, what is ahead of us, what cannot be predicted and explored in any available way. It is a domain of realized plans as well as of surprise. It combines expected and unexpected changes, gives rise to hopes and fears. When we open our eyes to the future and step beyond the limits of our existence, it becomes even more difficult to grasp. This is a result of theoretical and methodological deficits experienced in studies on psychological time (Zimbardo and Boyd, 1999), particularly when the subject is approached from such a wide angle.

The studies on the future that stem from the time perspective analysis (Frank, 1939; Lewin, 1942) were initiated by such thinkers as Fraisse (1967). He focused on the topic of time prediction. His work directed the researchers’ attention to temporality and its relationship with the functioning of individuals. Today, we have a multitude of studies that deal with the future time perspective and time orientation (but see Nuttin, 1984, 1985; Nurmi, 1991; Zaleski, 1994; Trempała and Malmberg, 1998; Nosal and Balcar, 2004; Diaz-Morales, 2006; Uchnast, 2006; Zimbardo and Boyd, 2008).

A problem which is rarely the subject of theoretical and empirical analyses is the future transcending the limits of temporality. In psychology it was Boyd and Zimbardo (1997, 2006) and Zimbardo and Boyd (2008), who attempted to operationalize and investigate the transcendental future. They explained the term as the time after life, i.e., from death to eternity (Boyd and Zimbardo, 1997, 2006; Zimbardo and Boyd, 2008). The transcendental future is associated with the capacity to believe in, think about and imagine the immortality (Seema et al., 2014). It encompasses the infinite time, which often reaches beyond our imagination and it is based on deep faith. Even if people make projections of that time (Boyd and Zimbardo, 2006; Zimbardo and Boyd, 2008; Timoszyk-Tomczak and Bugajska, 2012), they are of a different character than the projects created for the life below. What is essential is relevance rather that realism and subjectively perceived probability of success (Zaleski, 1988). Additionally, these projections last eternally, which gives it a different dimension (Zimbardo and Boyd, 2008). It also prevents their verification. Having created a vision of existence after death, one can only hope. In the context of the temporal future, the implementation of projects can be evaluated, improved, modified. In the context of the future beyond the mortality it is no longer possible to experience success or failure.

In psychology there are also other notions that refer to crossing the limits of earthly existence, such as spirituality, transcendence, transcendent wisdom or gerotranscendence. Spirituality is a concept of a complex structure whose components are such less abstract notions as hope, the meaning of life, forgiveness or a spiritual perspective (Reed and Rousseau, 2007). The spiritual perspective refers to the belief in something that cannot be directly experienced, something that is not subject to devaluation and that does not have to but may encompass religious experience (Reed and Larson, 2006). Transcendence and transcendent wisdom are also associated with spirituality (Straś-Romanowska, 2001; Heszen-Niejodek and Gruszczyńska, 2004). The process of transcendence taking place in late adulthood is a focal point in Tornstam’s (2005, 2011). It is founded on an assumption that human aging includes a potential to mature into a new outlook on and understanding of life. Gerotranscendence implies a shift in metaperspective, from a materialistic and rational view of the world to a more holistic and transcendent one (Tornstam, 2011). This includes the re-definition of one’s self, their relationship with other people and the world and, primarily, their concept of time. In such a context, their approach to the future beyond death may transcend their earlier understanding of this time. It may involve a new quality in integrating, experiencing and developing the time perspective.

Research on the future in old age (Timoszyk-Tomczak and Bugajska, 2012) led us to create a model for the future time perspective in late adulthood. This model was largely based on Nuttin’s (1985) and Lens (1986) future time perspective. At that time, we concluded that the future of older people was open and could be extended to include a transcendental aspect. The material we collected allowed us to conclude that individuals in late adulthood think about their future not only in their personal dimension, but also in the generational or even in the metaphysical one. Hence, we divided the elderly people’s future time perspective into personal (individual), generational and metaphysical parts. In the personal and generational future, we distinguished the temporal and transcendental aspects. Also, we linked transcendentalism with the metaphysical aspect, i.e., with the space-time transgression.

**FIGURE 1:**
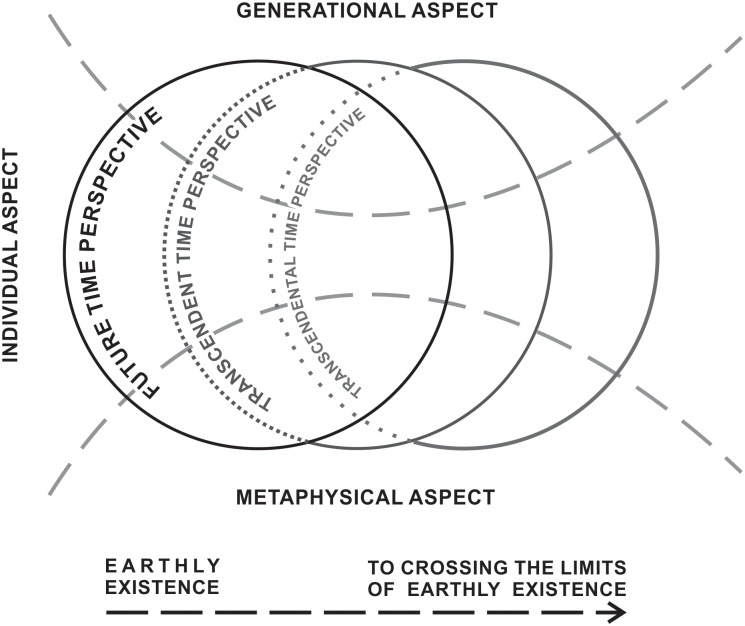
The model of the future time perspective together with the transcendental and transcendent future.

In the course of analyzing the data we had collected, doubts began to arise as to the realm beyond mortality which seemed heterogeneous. Faith in some form of life after death was not always associated with thinking about that time, but also with images of it. We were looking for explanations and distinctions in relation to that time. Tornstam’s concept of gerotranscendence; Tornstam (2005, 2011) was helpful in this regard. We focused on distinguishing between the transcendent and transcendental. We considered the transcendentality of the future primarily as faith and ruminating on time after death in terms of personal images such as meeting with loved ones or salvation rather than thinking about the future of generations to come or the universe. And we acknowledged the transcendence of the future as embracing both the temporality and what is beyond the temporality, present in the personal and generational dimension, and including the metaphysical aspect (Timoszyk-Tomczak and Bugajska, 2016). Transcendentalism and transcendence are not completely inseparable, but they have a different specificity. They can complement each other, but they can also function relatively independently. Transcendentality may be more related to cultural message, including attitudes toward religion, while transcendence refers rather to the internalization of values and models as well as spiritual development (Figure 1).

## Sources and Inspiration to Create Proposed Inventory

The theoretical model became the basis for the development of the inventory of the transcendental and transcendent future. Additional inspiration to develop the inventory was a 10-item Transcendental Time Perspective Inventory by Boyd and Zimbardo (1997, 2006) and Zimbardo and Boyd (2008). Measurements made with the use of this tool by the authors of this paper and other researchers (Ortuño et al., 2013; Seema et al., 2014; Maris Vázquez et al., 2016) show that the analysis of time after life to eternity can be a very interesting but difficult subject of studies. Zimbardo and Boyd’s tool focuses on the aspect of belief in life after death, particularly on the belief in some form of existence after death. It does not, however, measure how often a given person thinks about the time after death (Seema et al., 2014). Neither does it examine the future beyond individual existence.

The future beyond human life is a sensitive and complicated research subject. It can trigger one’s fear of death or engage their dysfunctional beliefs but, at the same time, it can reach beyond self-centeredness and elicit the process of transcendence. On the one hand it seems that the majority of humans think about death increasingly often as they are aging (Timoszyk-Tomczak and Bugajska, 2012). On the other hand, the nature of these thoughts may take various forms and refer to different issues. People can extend the future on a linear basis and simply create some projects for the time after death (Zimbardo and Boyd, 2008; Timoszyk-Tomczak and Bugajska, 2016), still remaining in the context of their lives below. They can also step outside their temporality and think about the future in the category of other generations, which is closer to the processes of gerotranscendence (Tornstam, 2011). Both these forms are not mutually exclusive but they can be partly separate ways to approach the future beyond mortal life. This is why it seems worthwhile to develop tools that will help examine these aspects of the future.

## Description of Proposed Tool

Subjectively perceived future refers to different levels, such as individual, generational or metaphysical future (Nilsson et al., 2003). It can embrace the mortal life or reach beyond death (Boyd and Zimbardo, 1997, 2006; Zimbardo and Boyd, 2008). The future transcending earthly life may become the area of individual projects – the plans whose characteristics differ from the temporal ones. On the other hand, it may mean re-definition of the self, the time and the hitherto existing context (Timoszyk-Tomczak and Bugajska, 2015).

The authors’ own studies on the elderly people’s future time perspective show that many people think about the time after their death and make predictions about it in various ways (Timoszyk-Tomczak and Bugajska, 2012). This thinking and the approach to the future understood in an open manner, i.e., as crossing the limits of human life, takes different forms. Sometimes it is restricted to ‘what is known’ or to what lies within the framework of one’s religious or non-religious beliefs, while sometimes it has a transcendental character, which means going beyond the way we have comprehended the reality and ourselves (Tornstam, 2011).

The transcendent and transcendental time perspective inventory examines the future that passes beyond the limits of personal future, beyond individual death and beyond the recognized, standard understanding of the self, the relationships and the world. It addresses the changes of qualitative and quantitative character. The inventory is composed of two sub-scales.

### The Transcendental Time Perspective

The transcendental time perspective refers to individual capacity to cross the limits of the earthly life, to mental traveling to eternity. It is first and foremost about believing in existence after death and imagining “what will happen to me after my death.” It includes “planning” things such as a meeting with relatives or salvation as well as the emotional attitude toward that time.

### The Transcendent Time Perspective

The transcendent time perspective is a holistic vision of life and death, devoid of the strong commitment of the “I.” A qualitative change in the perception of the future, both temporal and non-mortal. Apart from the personal aspect, it increasingly often includes the generational aspect, as well as the metaphysical one which is associated with the fate of the universe. It gives meaning to one’s own existence and allows one to reflect on their own life, but also on the life of other generations and the world. It makes the passing of human life easier to accept.

## Construction of Proposed Tool

The studies on the future time perspective of elderly people have shown that people in the late stage of adulthood often plan their earthly future and go beyond temporality while thinking about death and the time after death (Zimbardo and Boyd, 2008; Timoszyk-Tomczak and Bugajska, 2012). When analyzing the notions of spirituality, transcendence, transcendent wisdom or gerotranscendence, the distinction has been made into transcendent and transcendental future. The former is closer to gerotranscendence, whereas the latter – to comprehending the future after death in terms of the life below (Timoszyk-Tomczak and Bugajska, 2016). Having made the distinction between the two, an initial selection of a pool of 36 statements was made. A half of the statements refer to the transcendent future and another half – to the transcendental one. The questions were created on the basis of the prior conceptualization. After a content analysis and linguistic consultation, and following a discussion with experts 26 items were selected for the first version of the inventory.

### Survey Respondents and Procedure

The survey was carried out on two samples. An initial research procedure was run in 2017 in accordance with the Declaration of Helsinki. The written informed consent was obtained from the respondents of this study. The study was conducted in a group of 211 respondents (70% of women, 30% of men), with different levels of education and aging from medium to late adulthood (the average of 65). The selection for the sample was purposeful, as the tool is mainly designed for older people. The respondents were invited to the survey by trained students. The survey was of a group character, after presenting the purpose of the survey and instructions, the respondents filled in a questionnaire.

The second study was conducted in a group of 238 respondents (164 women and 67 men, 7 no gender data available), in the mean age of 66. As before, the selection of the sample was purposeful. The study was carried out in groups of people from the Senior Citizen’s Home, Third Age University and Social Welfare Home. Respondents were presented with the aim of the research and given a short instruction how to fill in the questionnaire.

## Psychometric Value of Inventory

In order to test the utility of the inventory and to assign the items to the sub-scales/sub-inventories an exploratory factor analysis EFA was performed in the first sample (211 respondents). The scree plot indicated the presence of two or three factors. The two- and three-factor versions were tested using oblimin rotation assuming the possibility of factors correlation. The two-factor analysis was more accurate. The total explained variance was 40%. Cronbach’s alpha for the first factor consisting of 15 items was 0.83, and for the second factor of 10 items it reached 0.88.

Next, internal accuracy was tested in the second sample (239), by means of the confirmatory factor analysis CFA (CMIN/df, RMSEA, GFI, AGFI, CFI, NFI, and AIC). The analyses performed on a model obtained following the factor analysis and free of modifications indicated a poor fit. After adding covariance paths, the indicators improved. The analysis revealed quite good fit indices of empirical data to the orthogonal factor model. Chi-square is relevant but when the distribution is inconsistent with normal distribution (to which this test is sensitive) and the sample is large, its value may rise. The remaining fit indices were good or acceptable (Table 1).

**Table 1:** Results of confirmatory factor analysis CFA.

Model	CMIN/df	RMSEA	GFI	AGFI	CFI	NFI	AIC
Model 2-czynnikowy ortogonalny	1.60	0.050	0.92	0.88	0.95	0.88	1719.73


**FIGURE 2:**
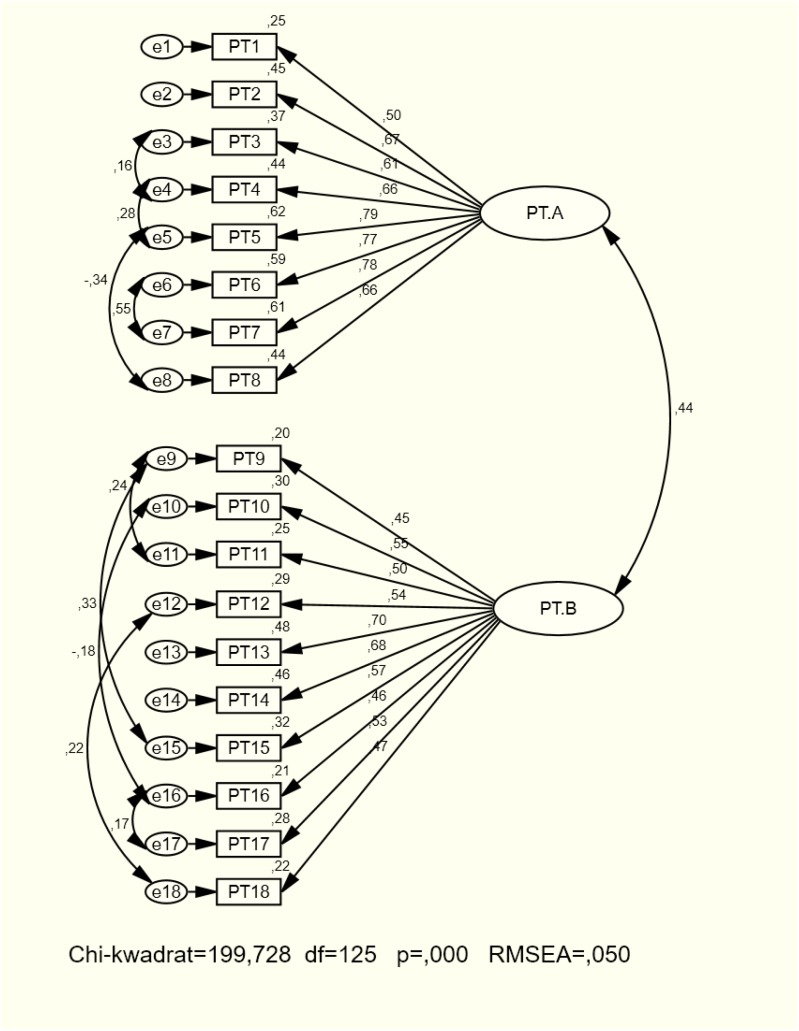
Factor structure of TTTPI.

In the factor A (transcendental future) covariances may indicate the consistency of items and faith in life after death is presumably the hidden variable. In the factor B (transcendent future), the future is associated with: the future of generations (9–11 and 9–15), the blurring of the boundary between life and death both in personal perspective and the universe (16–17), and acceptance of the indissolubility of life and death (12–18). Negative covariance (10–16) may reveal the complexity of transcendence, where the feeling of being a particle of the universe does not mean the blurring of the boundary between life and death (Figure 2).

The discriminative power of inventory items was evaluated on the basis of coefficients of correlation between the results calculated for each item and the result for the sub-scale as a whole. In the first sub-scale the correlation coefficients were stronger and ranged between 0.60 and 0.76, excluding Item 1 whose discriminative power was 0.43. In the second sub-scale the discriminative powers were weaker but still acceptable and ranged from 0.40 to 0.57. According to the internal consistency of the inventory measured with Cronbach’s alpha, the reliability of its final version (Appendix) was statistically satisfactory (Table 2).

**Table 2:** Characteristics of individual TTTPI items and of scale validity.

Transcendental time perspective		Transcendent time perspective	
	
Inventory item	Discriminative power	Inventory item	Discriminative power
1	0.43	9 (16)	0.51
2	0.61	10 (17)	0.44
3	0.60	11 (18)	0.42
4	0.68	12 (20)	0.48
5	0.75	13 (21)	0.57
6	0.73	14 (22)	0.52
7	0.76	15 (23)	0.56
8	0.60	16 (24)	0.41
		17 (25)	0.53
		18 (26)	0.40
Reliability of scales			

Transcendental time perspective		Transcendent time perspective	
	
0.88		0.81	


The psychometric properties of the inventory are satisfactory and make it a useful research tool in the studies on the time perspective after life and on the process of going beyond the present understanding of time (including the future), of the meaning of life as well as of human relationships with other people and the world.

## External Accuracy

The external accuracy of the inventory was tested with the use of the scale of Krok’s religious meaning system Krok’s (2009), Trapnell and Campbell’s rumination-reflection questionnaire Trapnell and Campbell’s (1999) adapted to Polish conditions by Radoń (2014), Zimbardo and Boyd’s short-form Time Perspective Inventory Zimbardo and Boyd’s (1999) adapted by Cybis et al. (2012) as well as Piotrowski, Skrzypińska and Żamojtel-Piotrowska’s spiritual transcendence scale (2013).

Multidimensional analyzes between the time perspective and religiosity were carried by Łowicki et al. (2018). Their study indicates that positive past and future dimension are of special importance for religiousness. Religious content helps people to explain and interpret reality in terms of meaning and purpose. For many religiously conscious people, religious beliefs manifest a desire to understand their position in relation to the world and other people (Krok, 2014). It was therefore decided to investigate the relationship between the religious meaning system and the transcendental and transcendent future. The scale of Krok’s religious meaning system Krok’s (2009) is used to measure religiousness in terms of meaning and contains two sub scales: religious orientation and religious sense. The first one refers to the understanding of the world and one’s own life, while the second to the interpretation of life in terms of meaning and purpose. The study was conducted on a group of 308 people (217 women and 91 men), aged 16–86, mean age 25. The results of the correlation analysis showed strong, significant links between the transcendental future and religious orientation and religious sense, as well as with the scale as a whole. We also found weaker correlations between the transcendent future and sub scales and the overall result of the scale of the religious meaning system. This is in line with the assumption that the transcendental time perspective is linked more to faith and personal future, while the transcendent time perspective goes beyond religious beliefs and has a more holistic character (Table 3).

**Table 3:** Interrelations between TTTPI and SRMS (*N* = 308).

Scales	Scale of orientation	Scale of sense	Total
Transcendental time perspective	0.591^∗∗^	0.617^∗∗^	0.623^∗∗^
Transcendent time perspective	0.308^∗∗^	0.274^∗∗^	0.299^∗∗^


*^∗^*p* < 0.05; ^∗∗^*p* < 0.01.*

Additionally, we compared the indicators of the variables of transcendental and transcendent time perspectives in the group of practicing (141) and non-practicing (49) believers in a group of 180 respondents (130 women, 40 men) aged 60–85. Due to the disproportion in the size of the groups, the non-parametric Mann-Whitney U test was applied. The results showed that in the group of practicing believers the indicator of transcendental time perspective was higher than the group of non-practicing believers. There were no statistically significant differences in the transcendent time perspective indicator. The obtained results are consistent with the assumptions concerning the comprehension of the transcendental and transcendent future (Table 4).

**Table 4:** Characteristics of non-practicing believers (*N* = 49) and practicing believers (*N* = 141) in TTP (factors) – comparative analysis with non-parametric Mann-Whitney *U-*test.

	Rank sum *practicing believers*	Rank sum *practicing believers*	*U*	*Z*	*p-*level	*Z* corrected	*p-*level
Transcendental TP	3067.50	15077.50	1842.50	-4.86	0.000	-4.87	0.000
Transcendent TP	4179.50	13965.50	2954.50	-1.51	0.132	-1.51	0.131


The Rumination-Reflection Questionnaire was chosen because of the assumption that the way of thinking about the future beyond mortality may change. Ruminations may be related to a transcendental time perspective based on faith and personal dimension of time, while reflection is more related to a transcendental time perspective, which is associated with the meaningfulness of human life. The rumination and time perspective tests were conducted in a group of 179 participants (131 women and 48 men) aged 58–96, the average of 68. The choice of subjects in their late adulthood was conditioned by the fact that the inventory was designed to test the transcendent and transcendental time perspective which referred to the perspective of the end of life, death and transcendence. The rumination questionnaire is used to examine its two aspects: positive and negative. The positive aspect relates to reflection and lies in openness to experience, while the negative one relates to rumination, i.e., non-adaptive thoughts, opinions and emotions, and stems from neurotic characteristics (Radoń, 2014). The analyses of correlation showed a weak (but relevant) positive relation between the transcendental time perspective and rumination. They also indicated a medium and strong positive correlation of the transcendent time perspective and reflection (Table 5). Those findings may be linked to the very nature of both scales that measure the attitude to the future beyond the present life. Transcendental future refers primarily to frequent thoughts about one’s own death, to believing in what is beyond death and, finally, to an emotional attitude which is probably less functional and inhibits adaptation. Therefore, it seems that aggravated rumination that is not associated with reflection captures the essence of this kind of reference to the time perspective reaching beyond present life. The transcendent future relates to changes based on modified understanding of reality and it is associated with personal development, which in turn indicates the need of open-minded perception of the future after death. Its links with reflection and rumination reveal a particular character of this attitude to the time beyond temporality.

**Table 5:** Inter-correlations between TTTPI and RRQ (*N* = 179).

Scales	Rumination	Reflection
Transcendental time perspective	0.162^∗^	0.127
Transcendent time perspective	0.218^∗∗^	0.385^∗∗^


*^∗^*p* < 0.05; ^∗∗^*p* < 0.01.*

Other relationships were found in the course of the correlation analyses using the Polish adaptation of Zimbardo and Boyd’s time perspective inventory. The absence of relevant correlations with the transcendental future indicates the discriminative accuracy of the tool (Table 6). The transcendental future shows the particular character of this way of perceiving time as it is not an element of the time perspective, being rather linked with believing in and focusing on what is going to happen after death. The transcendent future is related with the past, both negative and positive, with the hedonistic present as well as with the future. The correlation is the strongest with the hedonistic present which in the late adulthood means not only concentrating on pleasures, but also mindfully appreciating the life here and now (Bugajska and Timoszyk-Tomczak, 2014). This can also indirectly refer to life satisfaction that grows along with the process of gerotranscendence (Tornstam, 2011). The transcendent future is about perceiving time in its various dimensions and seems to extend the time perspective by the time after death.

**Table 6:** Inter-correlations between TTTPI and TPI (*N* = 179).

Scales	Positive past	Negative past	Hedonistic present	Fatalistic present	Future
Transcendental time perspective	0.093	0.083	0.011	0.100	-0.051
Transcendent time perspective	0.169^∗^	0.187^∗∗^	0.275^∗∗^	0.121	0.148^∗^


*^∗^*p* < 0.05; ^∗∗^*p* < 0.01.*

The study with the use of the transcendence scale (Piotrowski et al., 2013) covered 53 subjects (30 women and 23 men) aged 50–87, the average of 65. The transcendence scale is an operationalization of Piedmont’s spiritual transcendence theory Piedmont’s (1999; 2001) and consists of two sub-scales: proper transcendence and spiritual openness. The proper transcendence is about finding fulfillment in prayer, faith in life as a whole, the sense of bonding with other people that transcends death as well as about ideological zeal. The spiritual openness is about tolerating paradoxes, or the capacity to think many, not mutually exclusive ways, about non-judgmental approach to other lifestyles, rejoicing every moment and about thankfulness (Piotrowski et al., 2013). Inter-correlations indicate medium positive relations of the transcendent future and the transcendental future with the proper transcendence, which may imply that each of these dimensions includes the aspect of faith, thoughts and emotions referring to the time after death. However, only the transcendent future is connected with the spiritual openness which in turn gives broader perception of not only of personal freedom but also of the future of the world (Table 7).

**Table 7:** Inter-correlations between TTTPI and STS (*N* = 53).

Scales	Proper transcendence	Spiritual opennnes
Transcendental time perspective	0.542^∗∗^	-0.088
Transcendent time perspective	0.510^∗∗^	0.422^∗∗^


*^∗^*p* < 0.05; ^∗∗^*p* < 0.01.*

The obtained results slightly better show the character of the transcendental future that refers mainly to some form of life after death and includes an element of ‘planning’ what can happen beyond the current life. The transcendent future is of a more open nature as it is associated with thinking in the context of generations, a sense of life and its appreciation.

## Discussion

The analyses of the subjective approach to time, that are often based on studies on the time perspective, temporal orientation or attitude to time, suffer from the lack of proper tools to measure the attitude to the future transcending the current life. Theoretical analyses and the attempts to tap into the problem show deficiency and inconsistency symptoms in this matter (Zimbardo and Boyd, 2008; Ortuño et al., 2013; Seema et al., 2014; Maris Vázquez et al., 2016). Therefore, it seems essential to create such tools. The statistical analyses quoted above have proven that the proposed transcendental and transcendent time perspective inventory is an instrument that meets the requirements of reliability and accuracy. It is characterized by satisfactory internal coherence and rather good theoretical convergence. The confirmatory factor analysis has corroborated the validity of the chosen dimensions of the future beyond temporality. Therefore, it appears that the inventory is a good method for examining slightly differing attitudes to this time perspective: the transcendental future relating to faith in life after death, and the transcendent future that is focused on the man’s place in the universe, on the search for meaning and acceptance of the sense of existence. The inventory can be used in studies on adults, especially the older ones. It does not have a clinical character and requires relatively good cognitive performance, i.e., it is designed for individuals without clear deficits that can inhibit understanding the reality around them.

The performed analyses showed have shown strong links with the religious system of meanings, both with religious orientation and religious sense. They also revealed that believers score higher on the scale of transcendental future than non-believers. What is more, they also indicate the weakness of correlations between the transcendental future and a propensity to non-adaptive rumination, no links with the time perspective dimensions and a medium relationship with the proper transcendence. This indicates a specific nature of the transcendental future which is primarily associated with believing in any form of life after death. It is a way of thinking about the future beyond the limits of life that expresses the fear of future events, the recurrent contemplation of death and the lack of acceptance of the order of life. On the other hand, the transcendent future is more weakly correlated with the religious system of meanings as well as with rumination and, even to a larger extent, with reflection which has an adaptive character. It is also connected with diverse aspects of the time perspective, with the proper transcendence and spiritual openness. This may mean that the domination of this kind of thinking about the time after death is a part of the process of gerotranscendence (Tornstam, 2005, 2011). It encompasses stronger appreciation of what life is bringing as well as the openness to redefining one’s own experience. It does not exclude negative emotions and attitudes, but opens up to appreciation and to the search for meaning.

The study findings are merely an attempt to broaden the views on the time perspective and its specific character when it reaches beyond current life. It is a subject that pertains to existential fears and to the mystery of life, thus exceptionally difficult to explore. It would be interesting to examine the relationships of the transcendent and transcendental futures with the dimensions of personality and with religious attitudes. It would also be worthwhile to analyze the future beyond present life juxtaposed with mystical experience and a range of other aspects of human functioning.

On a practical level, the tool for measuring the attitude to the future after life can be used to support people who are at the end of their existence. Having learnt their approach to the time after life, it will be easier to effectively support them and their carers who often feel helpless when facing pain and fear of death.

## Author Contributions

CT-T contributed to preparation and development. BB contributed to consultation.

## Conflict of Interest Statement

The authors declare that the research was conducted in the absence of any commercial or financial relationships that could be construed as a potential conflict of interest.

## APPENDIX Transcendental and Transcendent Time Perspective Inventory

Read and decide to what extent the questions below are in line with your opinions, feelings and everyday practices. There are no good or bad answers here. What is important is what you think and feel. Mark your answer on the scale 1–5 and put in the number in a square next to the question. Choose the answer:

1 – I totally disagree, 2 – I disagree, 3 – I neither disagree nor agree, 4 – I agree, and 5 – I totally agree.

1. I think about the time after my death.
2. My future is not only what is temporal, but also what is beyond temporality.
3. I envision my life as eternal.
4. I believe that human life takes a different form after death.
5. I believe that there is something out of our body that will preserve after our death.
6. I believe that after death I will meet people close to me.
7. I think about meeting a Superior Being after my death.
8. I have my own important “goals-values” that I would like to realize after my death.
9. (16) When I am thinking about the future, I am thinking about future generations.
10. (17) I have a sense of being a part of the stream of life, what has been and what will be.
11. (18) I feel a bond with past and future generations.
12. (20) When I am thinking about the end of life and beyond, I feel acceptance. Gdy myślê o kresie życia i tym co poza nim to czuje akceptacjê.
13. (21) For me the future is about finding the sense of existence.
14. (22) For me thinking about the future is associated with thinking about the role of the man in the universe.
15. (23) When I am thinking about the future, I start thinking about people who will be living after me.
16. (24) When I am thinking about life and death, the boundaries between these two are blurred.
17. (25) For me the future is about crossing the boundaries of not only my own life but of the universe.
18. (26) For me the time of life and the time of death are inseparable.
